# The Human-Milk Oligosaccharide Profile of Lactating Women in Dhaka, Bangladesh

**DOI:** 10.1093/cdn/nzab137

**Published:** 2021-11-13

**Authors:** Lisa G Pell, Eric O Ohuma, Chloe Yonemitsu, Miranda G Loutet, Tahmeed Ahmed, Abdullah Al Mahmud, Meghan B Azad, Lars Bode, Daniel E Roth

**Affiliations:** Centre for Global Child Health and Child Health Evaluative Sciences, Research Institute, The Hospital for Sick Children, Toronto, Canada; Centre for Global Child Health and Child Health Evaluative Sciences, Research Institute, The Hospital for Sick Children, Toronto, Canada; Maternal, Adolescent, Reproductive and Child Health (MARCH) Centre, London School of Hygiene and Tropical Medicine, London, United Kingdom; Department of Pediatrics and Larsson-Rosenquist Foundation Mother-Milk-Infant Centre of Research Excellence, University of California San Diego, La Jolla, CA, USA; Centre for Global Child Health and Child Health Evaluative Sciences, Research Institute, The Hospital for Sick Children, Toronto, Canada; Dalla Lana School of Public Health, University of Toronto, Toronto, Canada; Nutrition and Clinical Services Division, International Centre for Diarrhoeal Disease Research, Bangladesh, Dhaka, Bangladesh; Nutrition and Clinical Services Division, International Centre for Diarrhoeal Disease Research, Bangladesh, Dhaka, Bangladesh; Manitoba Interdisciplinary Lactation Centre, Children's Hospital Research Institute of Manitoba, Department of Pediatrics and Child Health, University of Manitoba, Winnipeg, Canada; Department of Food and Human Nutritional Sciences, University of Manitoba, Winnipeg, Canada; Department of Pediatrics and Larsson-Rosenquist Foundation Mother-Milk-Infant Centre of Research Excellence, University of California San Diego, La Jolla, CA, USA; Centre for Global Child Health and Child Health Evaluative Sciences, Research Institute, The Hospital for Sick Children, Toronto, Canada; Dalla Lana School of Public Health, University of Toronto, Toronto, Canada; Department of Pediatrics, University of Toronto and Hospital for Sick Children, Toronto, Canada; Department of Nutritional Sciences, University of Toronto, Toronto, Canada

**Keywords:** human-milk oligosaccharides, vitamin D, Bangladesh, secretor status, breast milk

## Abstract

**Background:**

Human-milk oligosaccharides (HMOs) are an abundant component of human milk that have health-related effects on breastfeeding infants. Since variation in HMO composition can be explained by maternal and environmental factors, understanding the diversity in HMOs across settings and identifying context-specific factors associated with HMO abundances is important.

**Objectives:**

The aim was to describe the HMO profile of Bangladeshi women and to estimate the effect of maternal vitamin D supplementation on HMO composition.

**Methods:**

In a cross-sectional analysis of data and samples from the Maternal Vitamin D for Infant Growth trial in Dhaka, Bangladesh (clinicaltrials.gov; NCT01924013), 192 participants were randomly selected including 96 from each of the placebo and highest-dose vitamin D supplementation groups. In mid-feed breast milk samples collected at a mean (±SD) postpartum age of 93 ± 7 d, absolute and relative abundances of 19 HMOs were analyzed by HPLC. “Secretors” were defined as participants with 2′fucosyllactose concentrations >350 nmol/mL. Associations between HMO concentrations and selected maternal or environmental factors were estimated by multivariable linear regression, adjusting for vitamin D group allocation and secretor status. HMO profiles of Bangladeshi women were compared with data from other international cohorts.

**Results:**

Overall, 34% (65/192) of participants were nonsecretors. Secretor status was associated with the concentrations of total HMOs and 79% (15/19) of individual HMOs. Vitamin D supplementation did not affect the total or individual concentration of any measured HMO. 3-Fucosyllactose concentration was significantly higher in breast milk samples collected in December to February compared with samples collected in March to May. HMO composition was similar to other previously reported cohorts.

**Conclusions:**

The HMO profile of Bangladeshi women is predominantly determined by secretor status. Context-specific HMO data may improve understanding of the effects of HMOs on the infant microbiome and health and guide the development of HMO-containing interventions.

## Teaser

Human-milk oligosaccharide (HMO) profiles of Bangladeshi women are similar to those described in other countries. Vitamin D supplementation did not affect the Bangladeshi HMO profile.

## Introduction

Human-milk oligosaccharides (HMOs) are an abundant and biologically important nonnutritional component of human milk. Numerous functionalities have been attributed to HMOs, including their ability to influence the composition of early-life intestinal microbiota by promoting the growth of beneficial bacteria (1–3), inhibit host cell–pathogen interactions that are necessary for pathogenesis (4, 5), impact the innate and adaptive immune response (6), exert anti-inflammatory effects (7), promote optimal brain structure and function (8), and influence body composition and growth during infancy and childhood (9, 10). These biological effects of each HMO are closely linked to its particular molecular structure (11).

While more than 150 unique HMO structures have been reported (12), greater than 90% of the total HMO concentration in mature human milk is attributed to fewer than 20 different HMOs (13). However, there are important within- and between-individual variations in the concentrations and overall composition of HMOs. An individual woman's HMO concentrations vary throughout lactation stage, with the majority of HMO concentrations being higher in colostrum compared with mature milk (14, 15). Substantial between-women variations in HMO profiles are attributable to mutations in the gene that encodes α(1–2)fucosyltransferase 2 (FUT2) (16). A minority of women, categorized as nonsecretors, do not express active FUT2 and consequently their milk has very low concentrations of α(1–2)fucosylated HMOs, such as 2′fucosyllactose (2′FL) or lacto-N-fucopentaose (LNFP) 1 (LNFP1). While FUT2 activity partly explains between-women variation in HMOs, variation in HMO composition within secretor groups (17) indicates the influence of other determinants of HMO concentrations. HMO profiles were reported to vary across 12 geographically distinct populations that spanned urban and rural environments in 9 countries and 4 continents (17, 18). In addition, maternal factors, including age (18), weight (18), BMI (9, 18, 19), ethnicity (17), parity (17), and diet (20), have been shown to be associated with concentrations of some HMOs. Environmental factors, including seasonality, may also influence HMO composition (17); for example, in The Gambia, total HMO abundance was significantly higher in milk collected during the dry season compared with the wet season (21). Similarly, in a multi-ethnic Canadian cohort, the concentrations of some individual HMOs varied by season (17). Vitamin D status varies by season in Canada (22), although there is no known mechanism by which vitamin D influences HMO biosynthesis.

Notably, South Asian women have not been included in international comparisons of HMO composition published to date. We aimed to describe the HMO profile of human milk collected from 192 women in Dhaka, Bangladesh, overall and by secretor status. By leveraging the randomized design of the Maternal Vitamin D for Infant Growth (MDIG) placebo-controlled trial, we also aimed to estimate the effect of maternal vitamin D supplementation on HMO composition and to provide insight into a potential mechanism by which seasonality may influence HMO profile.

## Methods

### Design

Data and specimens were drawn from the MDIG trial (23), a randomized, double-blind, placebo-controlled, dose-ranging trial of maternal vitamin D supplementation during pregnancy and lactation in Dhaka, Bangladesh (clinicaltrials.gov; NCT01924013). In MDIG, a total of 1300 women in their second trimester of pregnancy (17 to 24 completed gestation weeks) were enrolled and randomly assigned to 1 of 5 parallel treatment groups. Participants received either 0 IU/wk (placebo), 4200 IU/wk, 16,800 IU/wk, or 28,000 IU/wk of vitamin D from enrollment until birth and 0 IU/wk from birth until 6 mo postpartum, or 28,000 IU/wk from enrollment until 6 mo postpartum. Detailed methods and findings from the MDIG trial have been published elsewhere (23, 24). The MDIG protocol was approved by the ethical review committees at the International Centre for Diarrhoeal Disease Research, Bangladesh (icddr,b) (Institutional Review Board protocol no. PR-13055) and the Hospital for Sick Children, Canada (Research Ethics Board no. 1000039072), and all MDIG participants provided informed consent for the storage and future analysis of stored biological specimens. The use of secondary clinical data and stored specimens for this sub-study was separately approved by the Hospital for Sick Children, Canada (Research Ethics Board no. 1000061001).

### Setting

Participants were enrolled at the Maternal Child Health Training Institute (MCHTI), a public hospital in Dhaka city that provides care to pregnant women and children in its catchment population (24). MDIG participants included in this sub-study were enrolled between 18 March 2014 and 7 May 2017. Three-month postpartum breast milk sample collection and 12-mo postpartum maternal anthropometry measurements were performed at MCHTI.

### Participants

**FIGURE 1:**
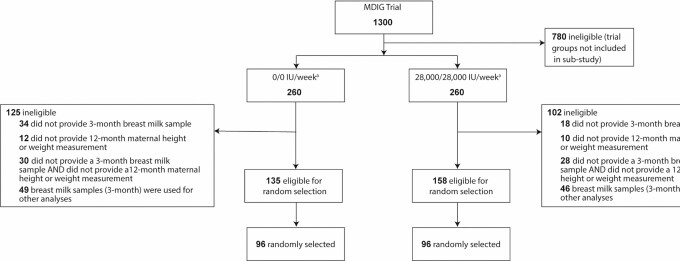
Flow chart of participant eligibility for inclusion and selection in the study. ^a^ MDIG trial group: dose of vitamin D3 supplementation given prenatally/postpartum.

Individuals were eligible to be included if they provided written informed consent to participate in the MDIG trial and consent for future use of biological specimens, were randomly assigned to receive either placebo or the highest dose of vitamin D (28,000 IU/wk) from enrollment until 6 mo postpartum, contributed a breast milk sample at approximately 3 mo postpartum that had not already been entirely utilized for other analyses, and provided a maternal height and weight measurement at approximately 12 mo postpartum. Among 1300 MDIG participants, 135 (52%) in the placebo and 158 (61%) in the high-dose prenatal/postpartum vitamin D groups were eligible for inclusion in this cross-sectional analysis (**Figure 1**). Among those meeting eligibility criteria for this sub-study, a simple randomization scheme was applied to select 96 participants from each of the placebo and vitamin D treatment groups. A formal sample size calculation was not performed. The inclusion of 96 samples per treatment group was made based on funding availability.

### Sample collection and laboratory measurements

Hand-expressed, mid-feed breast milk samples were collected at approximately 3 mo postpartum and stored in 1.5-mL aliquots at −70°C or colder until batched analysis (9). HMO analysis was conducted at the University of California San Diego using protocols that have been described elsewhere (18). In brief, an internal standard, raffinose, was added to human-milk samples (20 μL) to correct for sample loss and to allow for absolute quantification of 19 unique oligosaccharides. HMOs were separated from other milk components using high-throughput solid-phase chromatography over C18 (Hypercarb-96, 25 mg bed weight; Thermo Scientific) and Carbograph microcolumns (Hypersep-96 C18, 25 mg bed weight; Thermo Scientific) under a controlled vacuum manifold. The use of high-throughput microcolumns for this purpose has been previously validated (25). Isolated oligosaccharides were fluorescently labelled at their reducing end with the fluorophore 2-aminobenzamide (2AB; Sigma) in a 96-well thermocycler at 65°C for 2 h. The reaction was stopped by lowering the temperature to 4°C. Unreacted 2AB was separated from other reaction components by high-throughput solid-phase extraction using a silica microcolumn (Hypersep silica, 25 mg bed weight; Thermo Scientific). Labeled HMOs were analyzed by HPLC (Dionex Ultimate 3000; Dionex, now Thermo Scientific) on an amide-80 column (15 cm length, 2-mm inner diameter, 3-μm particle size; Tosoh Bioscience) buffered in 50 mmol/L ammonium formate–acetonitrile. The isolation of unique HMOs was performed at 25°C and monitored with a fluorescence detector at an excitation and emission wavelength of 360 nm and 425 nm, respectively. Standard column retention times of commercially available HMO standards (Sigma, Dextra, Elicityl) and a synthetic HMO library (26) combined with MS data generated on a Thermo LCQ Duo Ion trap mass spectrometer equipped with a Nano-ESI-source were used to annotate different HMOs. Absolute quantifications were calculated using HMO standard response curves for each of the 19 HMOs. The HMO detection limit was approximately 20 pmol, with a dynamic range between 20 and 5000 pmol; thus, milk samples were diluted on an as-needed basis. The total concentration of HMOs was calculated as the sum of the 19 individual HMO concentrations in nanomoles per milliliter. The relative amount of each HMO making up the total HMO concentration was also calculated. HMO-bound fucose was calculated on a molar basis, as described previously (20). Similar calculations were performed for HMO-bound sialic acid. Maternal secretor status was determined based on the absolute abundance of 2′FL in each milk sample using the natural breakpoint in the data (350 nmol 2′FL/mL) as the cutoff for secretor status determination. Women whose milk samples contained ≤350 nmol 2′FL/mL were categorized as nonsecretors.

### Statistical analysis

HMOs were grouped according to shared chemical characteristics that were previously defined (**Table 1**) (19). Differences in participant characteristics by secretor status were assessed by a chi-square test of association for categorical variables or Student's *t* test for continuous variables. Total, individual, and grouped HMO concentrations were described overall and by secretor status with geometric means (95% CIs), medians (IQRs), and ranges. Correlation matrix heat maps of Pearson correlation coefficients (*r*) were used to visualize and estimate correlations between individual HMO concentrations, overall and by secretor status. Pearson *r* ≥ 0.6 or *r* ≤ 0.6 was considered to represent at least moderate correlations between pairs of HMOs. Total and individual HMO concentrations were compared with values published for women from Canada, Ethiopia, The Gambia, Ghana, Kenya, Peru, Spain, Sweden, and the United States (17, 18). Variations in the proportion of women categorized as secretors by geographic location were compared without formal statistical testing.

**FIGURE 2:**
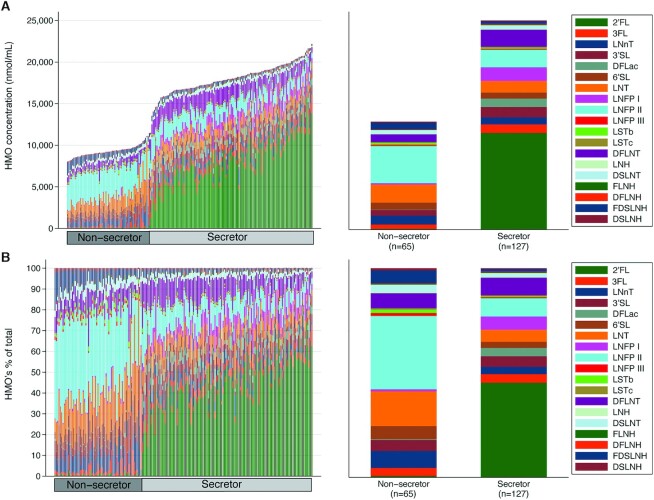
Total and individual abundances of 19 HMOs among 192 Bangladeshi participants, overall and by secretor status. (A) Total HMO content and absolute abundance of 19 HMOs for all 192 cohort participants, by secretor status. (B) Relative abundance of 19 HMOs (%) for all 192 cohort participants, by secretor status. Each column in the 2 graphs displaying HMO absolute and relative abundances represents 1 individual participant, ordered from lowest to highest concentration of 2′FL. DFLac, difucosyllactose; DFLNH, difucosyllacto-N-hexaose; DFLNT, difucosyllacto-N-tetrose; DSLNH, disialyllacto-N-hexaose; DSLNT, disialyllacto-N-tetraose; FDSLNH, fucodisialyllacto-N-hexaose; FLNH, fucosyllacto-N-hexaose; HMO, human-milk oligosaccharide; LNFP I, lacto-N-fucopentaose 1; LNFP II, lacto-N-fucopentaose 2; LNFP III, lacto-N-fucopentaose 3; LNH, lacto-N-hexaose; LNnT, lacto-N-neotetraose; LNT, lacto-N-tetrose; LSTb, sialyl-lacto-N-tetraose b; LSTc, sialyl-lacto-N-tetraose c; 2′FL, 2′fucosyllactose; 3FL, 3-fucosyllactose; 3′SL, 3′-sialyllactose; 6′SL, 6′-sialyllactose.

**TABLE 1:** HMOs measured in the study and their categorization based on chemical structure^1^

HMO	Structural categorization
3′-Sialyllactose (3′SL)	Sialylated lactose
6′-Sialyllactose (6′SL)	
2′-Fucosyllactose (2′FL)	Fucosylated lactose
3-Fucosyllactose (3FL)	
Difucosyllactose (DFLac)	
Sialyl-lacto-N-tetraose b (LSTb)	Non-fucosylated and sialylated
Sialyl-lacto-N-tetraose c (LSTc)	
Disialyllacto-N-hexaose (DSLNH)	
Disialyllacto-N*-*tetraose (DSLNT)	
Fucosyllacto-N-hexaose (FLNH)	Fucosylated and non-sialylated
Difucosyllacto-N-hexaose (DFLNH)	
Difucosyllacto-N-tetrose (DFLNT)	
Lacto-N-fucopentaose 1 (LNFP1)	
Lacto-N-fucopentaose 2 (LNFP2)	
Lacto-N-fucopentaose 3 (LNFP3)	
Lacto*-*N*-*hexaose (LNH)	Non-fucosylated and non-sialylated
Lacto-N-neotetraose (LNnT)	
Lacto-N-tetrose (LNT)	
Fucodisialyllacto-N-hexaose (FDSLNH)	Fucosylated and sialylated

1 HMO, human-milk oligosaccharide.

Due to right-skewing of HMO distributions, concentrations were log-transformed for all subsequent analyses. HMO concentrations were standardized on the log-scale by subtracting the HMO-specific mean and dividing by the HMO-specific SD (27, 28). Clustering of HMOs was based on computing a dissimilarity index using the Spearman correlation method (29, 30). Linear regression models were used to estimate the effect of vitamin D supplementation on HMO concentrations, adjusting for secretor status. Linear regression was also used to estimate associations of specific and total HMO concentrations with secretor status or other candidate predictors (whereby each HMO-factor association was considered in a separate model): maternal age, parity, highest level of education attained, BMI classification, preterm, infant feeding pattern at 3 and 6 mo, infant age at milk-sample collection, month of milk collection, and mode of delivery. All models were adjusted for vitamin D group allocation and secretor status. Parity was derived as the total number of pregnancies (including the pregnancy associated with the MDIG enrollment) minus the number of previous abortions/miscarriages. Breastfeeding pattern was derived based on the least optimal report of breastfeeding behavior that was documented in a given week over a 26-wk surveillance period. Given the relatively high proportion of infants who were given a prelacteal feed in the first week of life, infants who were not exclusively breastfed in week 1 but were exclusively breastfed in week 2 or were missing data for week 2, the breastfeeding pattern for the first week of life was defined as exclusively breastfed. Months were grouped as December to February, March to May, June to August, and September to November. Statistical significance was considered at the 5% level, and where multiple comparisons were performed, we accounted for the multiplicity of comparisons using the Holm procedure. All analyses were performed using Stata version 15 (StataCorp) and R statistical software version 4.0.3 (R Foundation for Statistical Computing).

## Results

### Participant characteristics

Participant characteristics are summarized in **Table 2**. Overall, 34% (*n* = 65) of participants were categorized as nonsecretors (**Figure 2**A). Breastfeeding pattern by 6 mo of age differed between nonsecretors and secretors (23% vs. 15%, exclusive; 8% vs. 9%, predominant; 57% vs. 69%, partial; 5% vs. 7%, none or discontinued; 8% vs. 1%, unable to classify) after adjusting for vitamin D status. However, breastfeeding pattern by 3 mo of age was not associated with secretor status (Table 2). None of the other factors (i.e., maternal age, parity, maternal education, maternal occupation, BMI, mode of delivery, postnatal age at milk collection, gestational age at birth, preterm birth) were significantly associated with secretor status (Table 2).

**TABLE 2:** Participant characteristics, overall and by secretor status

Characteristics	All women (*n* = 192)	Nonsecretors (*n* = 65)	Secretors (*n* = 127)	*P*
Maternal age, mean ± SD, y	23.8 ± 4.4	23.9 ± 4.9	23.7 ± 4.1	0.83
Age categories, *n* (%)				
18–24 y	110 (57.3)	40 (61.5)	70 (55.1)	0.18
25–29 y	58 (30.2)	14 (21.5)	44 (34.7)	
30–34 y	19 (9.9)	8 (12.3)	11 (8.7)	
35–38 y	5 (2.6)	3 (4.6)	2 (1.6)	
Parity, *n* (%)				
1	78 (40.6)	25 (43.1)	48 (39.0)	0.60
2	80 (41.7)	21 (36.2)	54 (43.9)	
3+	34 (17.7)	12 (20.7)	21 (17.1)	
Maternal education, *n* (%)				
No education	6 (3.1)	2 (3.1)	4 (3.2)	0.42
Primary incomplete	48 (25)	17 (26.2)	31 (24.4)	
Primary complete	25 (13)	5 (7.7)	20 (15.8)	
Secondary incomplete	74 (38.5)	24 (36.9)	50 (39.4)	
Secondary complete or higher	39 (20.3)	17 (26.2)	22 (17.3)	
BMI categories, *n* (%)				
Underweight	18 (9.4)	6 (9.2)	12 (9.5)	0.89
Normal weight	100 (52.1)	36 (55.4)	64 (50.4)	
Overweight	59 (30.7)	19 (29.2)	40 (31.5)	
Obese	15 (7.8)	4 (6.2)	11 (8.7)	
Maternal occupation, *n* (%)				
Homemaker	182 (94.8)	62 (95.4)	120 (94.5)	0.79
Other	10 (5.2)	3 (4.6)	7 (5.5)	
Gestational age at birth				
Mean ± SD, wk	39.0 ± 1.5	39.1 ± 1.4	39.0 ± 1.6	0.59
Preterm (<37 wk), *n* (%)	14 (7.3)	3 (4.6)	11 (8.7)	0.31
Mode of delivery, *n* (%)				
Vaginal	94 (49)	38 (58.5)	56 (44.1)	0.06
Cesarean	98 (51)	27 (41.5)	71 (55.9)	
Breastfeeding pattern up to 3 mo of age, *n* (%)				
Exclusive	106 (55.2)	37 (56.9)	69 (54.3)	0.69
Predominant	21 (10.9)	6 (9.2)	15 (11.8)	
Partial	52 (27.1)	16 (24.6)	36 (28.4)	
None or discontinued	6 (3.1)	2 (3.1)	4 (3.2)	
Unable to classify	7 (3.7)	4 (6.2)	3 (2.4)	
Breastfeeding pattern up to 6 mo of age, *n* (%)				
Exclusive	34 (17.7)	15 (23.1)	19 (15.0)	0.05
Predominant	16 (8.3)	5 (7.7)	11 (8.7)	
Partial	124 (64.6)	37 (56.9)	87 (68.5)	
None or discontinued	12 (6.3)	3 (4.6)	9 (7.1)	
Unable to classify	6 (3.1)	5 (7.7)	1 (0.8)	
Postnatal age at milk collection, mean ± SD, d	93.4 ± 6.9	93.1 ± 5.6	93.6 ± 7.5	0.68

### HMO profiles of lactating women in Dhaka, Bangladesh

Total HMO concentrations varied greatly among women (range: 8040.7 to 22,145.1 nmol/mL) (Figure 2A, **Supplemental Table 1**), with a geometric mean total concentration of 14,351.2 (95% CI: 13,699.6, 15,033.8) nmol/mL. Among nonsecretors, 2′FL accounted for 0.8% of the total HMOs in breast milk (compared to 46% in secretors) (Figure 2B), the absolute abundance of 2′FL ranged from 3.5 to 348.9 nmol/mL (compared to 1291.8 to 15,800.9 nmol/mL in secretors) (Figure 2A, **Supplemental Figure 1**), and the geometric mean total concentration of 2′FL was 41.2 (95% CI: 31.6–53.7) nmol/mL [compared to 7768.9 (95% CI: 7241.2–8335.1) nmol/mL in secretors] (Figure 2A, Supplemental Table 1). Low absolute and relative abundances of 2′FL explained the overall lower concentration of total HMOs among nonsecretors compared with secretors (Figure 2A, Supplemental Table 1). Some individual HMOs were present in higher relative amounts in breast milk samples collected from nonsecretors compared with secretors (Figure 2B; Supplemental Figure 1). For example, LNFP2 accounted for 35% of the total HMOs in breast milk collected from nonsecretors compared to 8.5% in secretors (Figure 2B; Supplemental Table 1). Among secretors, the total HMO concentration ranged from 11,458.5 to 22,145.1 nmol/mL, and the absolute concentrations of LNFP2, lacto-N-hexaose (LNH), fucodisialyllacto-N-hexaose (FDSLNH), and difucosyllacto-N-tetrose (DFLNT) each varied by more than 60-fold (Figure 2A, Supplemental Table 1). Among nonsecretors, the absolute concentrations of 2′FL, 3-fucosyllactose (3FL), difucosyllactose (DFLac), LNFP1, LNFP2, and FDSLNH each varied by at least ∼100-fold (Figure 2A, Supplemental Table 1). When HMOs were categorized based on their chemical structures, significant compositional differences were observed between secretors and nonsecretors (**Table 3**). For example, HMOs composed of fucosylated or sialylated lactose molecules contributed to a greater proportion of the total abundance of HMOs in the milk samples collected from secretors compared with nonsecretors (62% vs. 17%; *P* < 0.001).

**TABLE 3:** Absolute and relative abundances of HMO groups that have been combined based on chemical structure, overall and by secretor status^1^

	All (*n* = 192)				Secretors (*n* = 127)				Nonsecretors (*n* = 65)				
HMO	Geometric mean (95% CI)	Range (min–max)	Median (IQR)	%/mL	Geometric mean (95% CI)	Range (min–max)	Median (IQR)	%/mL	Geometric mean (95% CI)	Range (min–max)	Median (IQR)	%/mL	*P*
Fucosylated or sialylated lactose^2^	5399 (4661–6253)	663–18,933	9176 (1801 – 12,009)	53	10,837 (10,361 – 11,335)	4763–18,933	10,879 (9225 – 12,829)	62	1384 (1237–1548)	663–6424	1284 (1001–1810)	17	<0.001
Non-fucosylated and non-sialylated^3^	1760 (1646–1881)	265–7851	1786 (1359–2317)	13	1583 (1465–1709)	265–5118	1627 (1181–2076)	10	2164 (1930–2427)	541–7851	2147 (1714–2676)	26	<0.001
Fucosylated and non-sialylated^4^	4148 (3973–4330)	286–6666	4253 (3809–4819)	28	4244 (4072–4422)	1591–6666	4338 (3751–4864)	24	3966 (3590–4381)	286–5868	4178 (3822–4658)	45	0.14
Non-fucosylated and sialylated^5^	539 (514–566)	190–1536	541 (442–669)	4	510 (479–542)	190–1240	508 (409–627)	3	601 (560–645)	322–1536	609 (490–734)	7	0.001
Fucosylated and sialylated^6^	239 (208–274)	5–1433	271 (157–463)	2	170 (146–199)	5–562	193 (116–313)	1	464 (386–557)	14–1433	505 (349–702)	6	<0.001

1 Values are in nmol/mL unless otherwise indicated. *P* values were derived using *t* test for comparison of the log-transformed concentrations of grouped HMOs between secretors and nonsecretors. DFLac, difucosyllactose; DFLNH, difucosyllacto-N-hexaose; DFLNT, difucosyllacto-N-tetrose; DSLNH, disialyllacto-N-hexaose; DSLNT, disialyllacto-N-tetraose; FDSLNH, fucodisialyllacto-N-hexaose; FLNH, fucosyllacto-N-hexaose; HMO, human-milk oligosaccharide; LNFP1, lacto-N-fucopentaose 1; LNFP2, lacto-N-fucopentaose 2; LNFP3, lacto-N-fucopentaose 3; LNH, lacto-N-hexaose; LNnT, lacto-N-neotetraose; LNT, lacto-N-tetrose; LSTb, sialyl-lacto-N-tetraose b; LSTc, sialyl-lacto-N-tetraose c; min–max, minimum–maximum; 2′FL, 2′fucosyllactose; 3FL, 3-fucosyllactose; 3′SL, 3′-sialyllactose; 6′SL, 6′-sialyllactose.

2 Calculated as 2′FL + 3FL + DFLac + 3′SL + 6′SL.

3 Calculated as LNT + LNnT + LNH.

4 Calculated as LNFP1 + LNFP2 + LNFP3 + DFLNT + FLNH + DFLNH.

5 Calculated as LSTb + LSTc + DSLNT + DSLNH.

6 Calculated as FDSLNH.

### Correlations and clustering of individual HMOs

**FIGURE 3:**
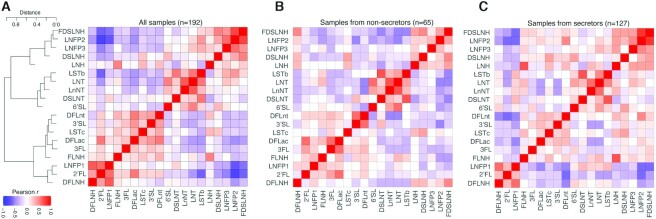
Correlation and clustering between individual HMO concentrations, (A) overall and (B, C) by secretor status. The range of colors represents the Pearson correlation coefficients (*r*), which describe the strength (darker the color, the stronger the correlation) and direction (red/positive vs. blue/negative vs. white/no correlation) of the correlation between pairs of individual HMO concentrations. The distance (or lack of similarity) between each node and cluster in the dendrogram (A) was based on a dissimilarity index using Spearman correlation. Data above and below the diagonal line are identical. DFLac, difucosyllactose; DFLNH, difucosyllacto-N-hexaose; DFLNT, difucosyllacto-N-tetrose; DSLNH, disialyllacto-N-hexaose; DSLNT, disialyllacto-N-tetraose; FDSLNH, fucodisialyllacto-N-hexaose; FLNH, fucosyllacto-N-hexaose; HMO, human-milk oligosaccharide; LNFP1, lacto-N-fucopentaose 1; LNFP 2, lacto-N-fucopentaose 2; LNFP 3, lacto-N-fucopentaose 3; LNH, lacto-N-hexaose; LNnT, lacto-N-neotetraose; LNT, lacto-N-tetrose; LSTb, sialyl-lacto-N-tetraose b; LSTc, sialyl-lacto-N-tetraose c; 2′FL, 2′fucosyllactose; 3FL, 3-fucosyllactose; 3′SL, 3′-sialyllactose; 6′SL, 6′-sialyllactose.

Among all participants, concentrations of 2′FL and LNFP1 as well as LNFP2 and FDSLNH clustered together (**Figure 3**A) and were positively correlated (Pearson *r* = +0.60 and *r* = +0.75, respectively). The concentration of 2′FL was negatively correlated with LNFP2, sialyl-lacto-N-tetraose b (LSTb), and FDSLNH (Pearson *r* = −0.80 to *r* = −0.63) (Figure 3A). Concentrations of 3FL, 3′-sialyllactose (3′SL), and sialyl-lacto-N-tetraose c (LSTc) did not correlate with the concentration of any other HMOs. Among secretors, the concentrations of 2′FL, LNFP2, and DFLNT were negatively correlated (Pearson *r* = −0.62 to *r* = −0.58) and the concentrations of lacto-N-neotetraose (LNnT) and lacto-N-tetrose (LNT) were positively correlated (Pearson *r* = +0.63) (Figure 3B). Among nonsecretors, both 2′FL and 3FL were positively correlated with DFLac (Pearson *r* = +0.58 and *r* = +0.58, respectively) (Figure 3C).

### Factors associated with HMO concentrations of lactating women in Dhaka, Bangladesh

**FIGURE 4:**
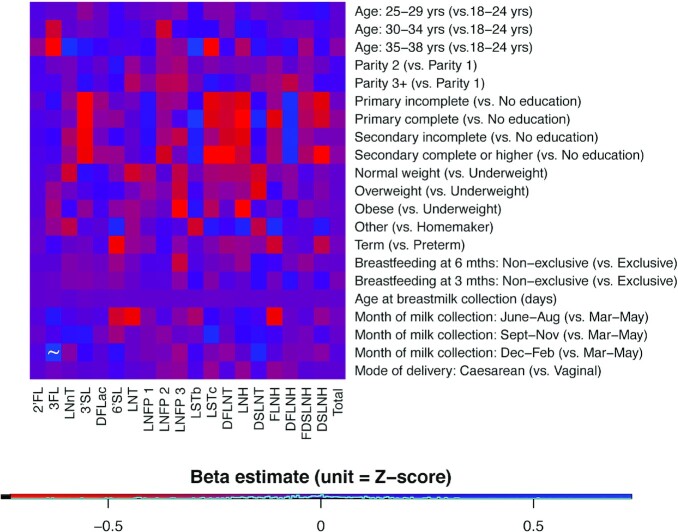
Multivariable analysis of selected maternal and environmental factors and HMO composition among a cohort of 192 Bangladeshi women. The range of colors represents the direction and magnitude of the estimated effect size; all log-transformed HMO values were standardized by subtracting the HMO-specific mean from each HMO value and dividing by the HMO-specific standard deviation (equivalent to a Z-score) to allow for comparisons across different HMOs in multiple regression analyses. A separate multiple linear regression model was used to estimate each HMO-factor association, adjusting for vitamin D group and secretor status. One variable was marked with the “∼” symbol to indicate statistical significance after accounting for multiple testing using the Holm procedure. DFLac, difucosyllactose; DFLNH, difucosyllacto-N-hexaose; DFLNT, difucosyllacto-N-tetrose; DSLNH, disialyllacto-N-hexaose; DSLNT, disialyllacto-N-tetraose; FDSLNH, fucodisialyllacto-N-hexaose; FLNH, fucosyllacto-N-hexaose; HMO, human-milk oligosaccharide; LNFP1, lacto-N-fucopentaose 1; LNFP2, lacto-N-fucopentaose 2; LNFP3, lacto-N-fucopentaose 3; LNH, lacto-N-hexaose; LNnT, lacto-N-neotetraose; LNT, lacto-N-tetrose; LSTb, sialyl-lacto-N-tetraose b; LSTc, sialyl-lacto-N-tetraose c; 2′FL, 2′fucosyllactose; 3FL, 3-fucosyllactose; 3′SL, 3′-sialyllactose; 6′SL, 6′-sialyllactose.

Vitamin D supplementation did not have an effect on total or individual HMO concentrations (**Supplemental Table 2**). Secretor status was significantly associated with total HMO concentration and the concentrations of 15 of 19 (79%) individual HMOs (Supplemental Table 2). Both negative and positive associations between secretor status and individual HMOs were observed (Supplemental Table 2). Month of milk collection (December–February vs. March–May) was significantly and positively associated with the concentration of 3FL (**Figure 4**). None of the other factors investigated were associated with total or individual HMO concentrations (Figure 4).

### Comparison of the HMO profile of women in Dhaka, Bangladesh, with other international cohorts

**FIGURE 5:**
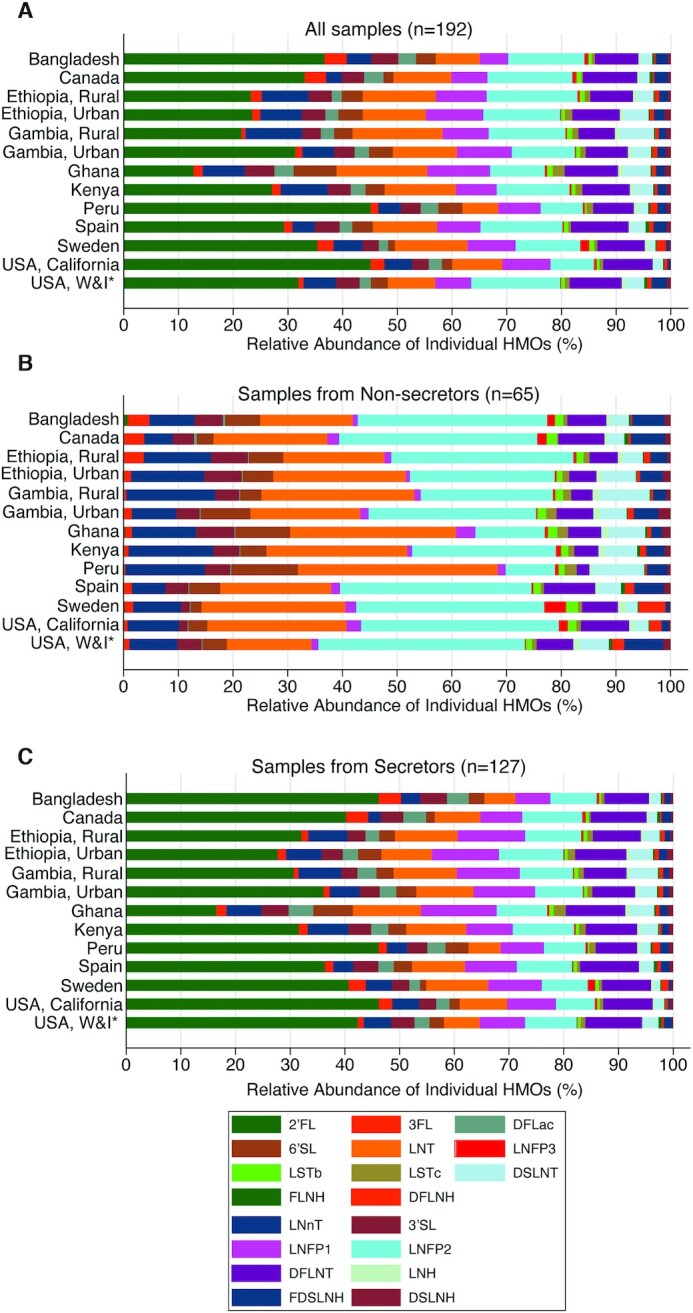
Variation in the relative abundances of 19 individual HMOs in breast milk collected from lactating women who reside in 13 different geographic locations worldwide, overall (A) and by secretor status (B, C). Data generated from women in the Canadian CHILD Cohort study were previously published by Azad et al. (17). Data generated from women in all other settings, with the exception of Bangladesh, were previously published by McGuire et al. (18) *“W&I” is used to describe a cohort from southeastern Washington and northwestern Idaho, USA. DFLac, difucosyllactose; DFLNH, difucosyllacto-N-hexaose; DFLNT, difucosyllacto-N-tetrose; DSLNH, disialyllacto-N-hexaose; DSLNT, disialyllacto-N-tetraose; FDSLNH, fucodisialyllacto-N-hexaose; FLNH, fucosyllacto-N-hexaose; HMO, human-milk oligosaccharide; LNFP1, lacto-N-fucopentaose 1; LNFP II, lacto-N-fucopentaose 2; LNFP III, lacto-N-fucopentaose 3; LNH, lacto-N-hexaose; LNnT, lacto-N-neotetraose; LNT, lacto-N-tetrose; LSTb, sialyl-lacto-N-tetraose b; LSTc, sialyl-lacto-N-tetraose c; 2′FL, 2′fucosyllactose; 3FL, 3-fucosyllactose; 3′SL, 3′-sialyllactose; 6′SL, 6′-sialyllactose.

The geometric mean of the total concentration of HMOs in lactating women in Dhaka, Bangladesh (14,351.2 nmol/mL), was comparable to average concentrations that have been previously reported for women in other cohorts worldwide (17, 18). The prevalence of nonsecretors in this cohort of Bangladeshi women (34%) was also similar to or somewhat higher than the proportional population of nonsecretors that have been previously reported from cohorts in rural and urban Ethiopia (35% and 22%, respectively), rural Gambia (35%), Ghana (32%), Spain (24%), Sweden (21%), southeastern Washington and northwestern Idaho in the United States (32%) (18), and Canada (28%) (17). Bangladesh had a higher proportion of nonsecretors compared to urban Gambia (15%), Kenya (19%), Peru (2%), and Hispanic women recruited in San Diego, California, in the United States (5%) (18). Overall, the relative contribution of each individual HMO among women in Bangladesh was similar to the distribution observed in other geographic settings (**Figure 5**A). However, nonsecretor Bangladeshi women appear to have a greater relative abundance of 2′FL in their milk compared with nonsecretors who reside in other geographic locations (Figure 5B). In addition, while the relative abundance of 3FL (4.1%) in breast milk collected from Bangladeshi secretor women was similar to the relative abundance of 3FL observed in breast milk collected from Canadian women (3.9%), 3FL made up a larger proportion of total HMO content in the breast milk from Bangladeshi women compared with breast milk collected from women in rural and urban Ethiopia (1.9% and 1.5%, respectively), rural and urban Gambia (0.8% and 1.2%, respectively), Ghana (1.7%), Kenya (1.6%), Peru (1.4%), Spain (1.5%), Sweden (3.0%), and southeastern Washington (0.9%) and northwestern Idaho (2.5%) in the United States (Figure 5C).

## Discussion

The total HMO concentration, relative abundance of each quantified HMO, and the proportion of secretors in this Bangladeshi cohort were similar to findings in several other international cohorts (17, 18). Within groups defined by secretor status, the relative abundance of individual HMOs among Bangladeshi women was, with few exceptions, also consistent with what has been reported for women in other geographic locations. Notably, nonsecretor Bangladeshi women appeared to have a greater relative abundance of 2′FL compared with nonsecretors from other geographic locations. This observation was unlikely to be related to variation in the timing of milk collection between cohorts; although the absolute concentration of 2′FL decreases over the course of lactation (31, 32) and has been shown to be 2 times lower in samples collected at 3 mo postpartum compared with the immediate postpartum period (32), the majority of samples being used as comparators were collected earlier (18) than the samples in this cohort, suggesting that this finding may be even more pronounced if earlier breast milk samples had been examined in the Bangladeshi cohort. Mutations in the *FUT2* gene that are responsible for the nonsecretor phenotype have been shown to vary between different ethnic populations (33–36). Genetic sequencing of the *FUT2* gene was not performed in this study, but it is possible that differences in the specific mutations between Bangladeshi nonsecretors and nonsecretors from different geographic locations may play a role in the different abundances of 2′FL observed. It is also possible that the relatively higher abundances of 2′FL observed in this study were an artifact of the threshold selected to define secretor status. In contrast to 2′FL, the greater relative abundance of 3FL in breast milk collected from Bangladeshi secretor women (4%) compared with breast milk collected from women in other international cohorts [ranging from 0.8% in rural Gambia and 0.9% in southeastern Washington, United States, to 2% rural Ethiopia and 3% in Sweden (18)] may potentially be explained by differences in lactation stage at the time of sample collection. The concentration of 3FL rises throughout lactation and has been shown to increase by almost 3-fold between day 2 postpartum and 3 mo postpartum (15, 18, 32).

Previous studies have shown that concentrations of DSLNT below a threshold of 241 nmol/mL are independently associated with an increased risk of developing necrotizing enterocolitis (NEC) in preterm infants (37, 38). Since rates of NEC among Bangladeshi preterm, very low birth-weight infants are between 11% and 14% (39, 40), knowledge of the proportion of Bangladeshi women with DSLNT concentrations below the protective threshold is important. In this cohort, 69 (8.5%) women had concentrations of DSLNT below 241 nmol/mL and, among these women, DSLNT concentrations ranged from 79 to 240 nmol/mL. There was no difference in the concentration of DSLNT between secretor and nonsecretor Bangladeshi women in this study.

As reported elsewhere (17, 18, 41), secretor status was a major determinant of both total and individual HMO concentrations among Bangladeshi women. We were otherwise unable to explain much of the variability in total, absolute, and relative HMO abundances based on the candidate predictors we studied. The concentration of 3FL was higher in breast milk samples collected in December to February compared with March to May, but this conflicted with previous observations in a multi-ethnic Canadian cohort of lower LNFP3 in the spring, higher LNnT and DSLNT in the spring, and lower 6′-sialyllactose (6′SL) in winter (17). By leveraging the randomized design of the MDIG trial, we were able to explore the effect of maternal supplementation with 28,000 IU vitamin D/wk, a micronutrient that can vary with season due to its endogenous production in the skin when exposed to UV radiation. The absence of any effect of a relatively high dose of supplemental vitamin D on total or individual HMO concentrations suggests that vitamin D status is unlikely to explain the seasonality of some HMO concentrations. In contrast to findings of other studies, parity (17, 42), maternal BMI (18), maternal age (17), and gestational age at delivery (43) were not associated with total or individual HMO composition among Bangladeshi women. Previous studies have shown that maternal diet is associated with HMO composition (20); however, direct evidence in support of the role of maternal diet in the seasonality of HMO composition is lacking. While maternal diet was not investigated as a candidate predictor variable in this cohort, future studies should aim to investigate the relation between season, maternal diet, and HMO composition.

A key limitation of this study was that breast milk samples were analyzed from only a single time point during lactation (3 mo postpartum), so an analysis of the effect of lactation stage on HMO composition in Bangladeshi women was not possible. In addition, the study population was recruited from 1 hospital that serves an urban community in Dhaka that may not be representative of Bangladesh in general. Greater variance in HMO profiles may have been observed by including women from more diverse socioeconomic and environmental settings.

It is well documented that HMOs promote the colonization and growth of beneficial bacteria, including some species of bifidobacteria (1) and members of the genus *Bacteroides* (3), and that the ability to metabolize particular HMOs varies between different species and strains of bacteria (44, 45). In addition, associations have been observed between maternal secretor status and milk microbiota (46) and maternal (47, 48) and infant (49) fecal microbiota. Therefore, knowledge of HMO profiles, and secretor status in particular, could inform the development of infant interventions that interact with the gut microbiota, such as prebiotics or synbiotics (probiotic/prebiotic combination), by providing exogenous HMOs to non-breastfed infants or breastfeeding infants whose mothers have relatively low abundances of specific HMOs. In fact, oligosaccharides that are identical to those present in human milk have recently been included in some commercially available infant formulas (50). However, given the wide variation in HMO profiles both within and between women, additional research is warranted to precisely define the amount and type(s) of exogenously added HMOs that could prove beneficial to infants.

In conclusion, the description of the HMO profile of lactating women in Bangladesh provides a basis for future research of HMOs in this setting and may guide the development of novel and targeted HMO-containing interventions.

## Supplementary Material

nzab137_Supplemental_Files

## Data Availability

Data described in the manuscript, code book, and analytic code will be made available upon request to the authors. De-identified individual participant data will be provided for use in secondary data analyses approved by an independent research ethics board, and data requestors will be required to sign a data access agreement.

## Notes

Supported, in whole or in part, by the Bill & Melinda Gates Foundation (OPP1066764 and OPP1187937). Under the grant conditions of the Foundation, a Creative Commons Attribution 4.0 Generic License has already been assigned to the Author Accepted Manuscript version that might arise from this submission. EOO was supported by the Child Growth and Development Fellowship Program at the Center for Global Child Health, Hospital for Sick Children.

Author disclosures: DER has received funding from the Bill & Melinda Gates Foundation (OPP1192472) to conduct clinical research on probiotics in Dhaka, Bangladesh. MBA holds a Tier 2 Canada Research Chair in the Developmental Origins of Chronic Disease at the University of Manitoba and is a Fellow in the Canadian Institutes for Advanced Research (CIFAR) Humans and the Microbiome Program. She receives research funding from the Canadian Institutes of Health Research, Allergy Genes and Environment (AllerGen) Network of Centers of Excellence, the Canadian Lung Association, Research Manitoba, the Canada Foundation for Innovation, the Bill & Melinda Gates Foundation, the Manitoba Children's Hospital Foundation, Prolacta Biosciences, Mitacs, CIFAR, the Garfield Weston Foundation, Health Data Research UK, and the Canadian COVID Immunity Task Force. She regularly speaks at conferences and workshops on infant nutrition, some sponsored by Prolacta Biosciences, and has spoken at a conference sponsored by AstraZeneca. She has contributed without remuneration to online courses on breast milk and the infant microbiome produced by Microbiome Courses. She serves in a volunteer capacity for the International Society for Research on Human Milk and Lactation and as a member of the National Academy of Sciences, Engineering, and Medicine Committee on Scanning New Evidence on the Nutrient Content of Human Milk. She has consulted for DSM Nutritional Products and serves on the Malaika Vx Scientific Advisory Board. LB is the Larsson-Rosenquist Foundation Chair of Collaborative Human Milk research, endowed by the Family Larsson-Rosenquist Foundation. He has received honoraria for speaking at national and international meetings and conferences supported by Medela, Prolacta, Abbott Nutrition, Mead Johnson, and Nestle Nutrition Institute. He is also a co-inventor on patent applications related to the use of human-milk oligosaccharides in preventing necrotizing enterocolitis and other inflammatory diseases. The other authors report no conflicts of interest. The funders played no role in the design, implementation, analysis, and interpretation of data.

Supplemental Figure 1 and Supplemental Tables 1 and 2 are available from the “Supplementary data” link in the online posting of the article and from the same link in the online table of contents at https://academic.oup.com/cdn/.

Abbreviations used: DFLac, difucosyllactose; DFLNH, difucosyllacto-N-hexaose; DFLNT, difucosyllacto-N-tetrose; DSLNH, disialyllacto-N-hexaose; DSLNT, disialyllacto-N-tetraose; FDSLNH, fucodisialyllacto-N-hexaose; FLNH, fucosyllacto-N-hexaose; FUT2, α(1-2)fucosyltransferase 2; HMO, human-milk oligosaccharide; LNFP1, lacto-N-fucopentaose 1; LNH, lacto-N-hexaose; LNnT, lacto-N-neotetraose; MCHTI, Maternal Child Health Training Institute; MDIG, Maternal Vitamin D for Infant Growth; NEC, necrotizing enterocolitis; 2AB, 2-aminobenzamide; 2′FL, 2′fucosyllactose; 3FL, 3-fucosyllactose.
