# Supplementary material for: The Human-Milk Oligosaccharide Profile of Lactating Women in Dhaka, Bangladesh
Source: Curr Dev Nutr. 2021 Nov 13;5(12):nzab137. doi: 10.1093/cdn/nzab137 (PMC8728024; doi:10.1093/cdn/nzab137)
Supplement: nzab137_Supplemental_Files [file nzab137_supplemental_files.zip › SupplementaryData_HMOcomposition_Bangladesh.docx]

**Supplementary data for:**

**The human milk oligosaccharide profile of lactating women in Dhaka, Bangladesh**

Lisa G. Pell^1^, Eric O. Ohuma^1,2^, Chloe Yonemitsu^3^, Miranda G. Loutet^1,4^, Tahmeed Ahmed^5^, Abdullah Al Mahmud^5^, Meghan B. Azad^6,7^, Lars Bode^3^, Daniel E. Roth^1,4,8,9*^

^1^Centre for Global Child Health and Child Health Evaluative Sciences, Research Institute, The Hospital for Sick Children, Toronto, Canada

^2^Maternal, Adolescent, Reproductive and Child Health (MARCH) Centre, London School of Hygiene and Tropical Medicine (LSHTM), London, UK

^3^Department of Pediatrics and Larsson-Rosenquist Foundation Mother-Milk-Infant Centre of Research Excellence, University of California San Diego, La Jolla, California

^4^Dalla Lana School of Public Health, University of Toronto, Toronto, Canada

^5^Nutrition and Clinical Services Division, icddr,b, Dhaka, Bangladesh

^6^Manitoba Interdisciplinary Lactation Centre (MILC), Children’s Hospital Research Institute of Manitoba, Department of Pediatrics and Child Health, University of Manitoba, Winnipeg, Canada

^7^Department of Food and Human Nutritional Sciences, University of Manitoba, Winnipeg, Canada

^8^Department of Paediatrics, University of Toronto and Hospital for Sick Children, Toronto, Canada

^9^Department of Nutritional Sciences, University of Toronto, Toronto, Canada

**List of Supplementary Tables & Figures**

Supplemental Table 1. Total and individual abundances of 19 HMOs among 192 Bangladeshi participants, overall and by secretor status.

Supplemental Table 2. The effect of vitamin D and secretor status on HMO concentrations among a cohort of 192 lactating Bangladeshi women.

Supplemental Figure 1. Total and individual abundances of 19 HMOs among 192 Bangladeshi participants, by secretor status.

**Supplementary Tables**

Supplemental Table 1. Total and individual abundances of 19 HMOs among 192 Bangladeshi participants, overall and by secretor status. The geometric mean (95%CI), range (min-max), median (IQR), %/ml, and between women variation in HMO concentrations are summarized for each of the 19 measured individual HMOs and total HMO, overall and by secretor status.

Due to the size of Supplemental Table 1, the document has been submitted as an Excel attachment called “Table1_SupplementaryData_v1_20210826”.

**Supplemental Table 2**. The effect of vitamin D and secretor status on HMO concentrations among a cohort of 192 lactating Bangladeshi women.

| **HMO** | **Characteristics** | **Number (%)** | **Difference in HMO concentration (log-nmol/ml)  (95% CI)** | **P-value** |
| --- | --- | --- | --- | --- |
| 2'FL | **HMO secretor status** |  |  |  |
|  | No | 65 (34) |  |  |
|  | Yes | 127 (66) | 5.241 (5.03, 5.45) | <0.001 |
|  | **Treatment allocation group** |  |  |  |
|  | Placebo | 96 (50) |  |  |
|  | 28000:28000 IU/week | 96 (50) | 0.020 (-0.18, 0.22) | 0.845 |
| 3FL | **HMO secretor status** |  |  |  |
|  | No | 65 (34) |  |  |
|  | Yes | 127 (66) | 1.267 (1.05, 1.49) | <0.001 |
|  | **Treatment allocation group** |  |  |  |
|  | Placebo | 96 (50) |  |  |
|  | 28000:28000 IU/week | 96 (50) | -0.069 (-0.28, 0.14) | 0.519 |
| LNnT | **HMO secretor status** |  |  |  |
|  | No | 65 (34) |  |  |
|  | Yes | 127 (66) | -0.178 (-0.34, -0.01) | 0.036 |
|  | **Treatment allocation group** |  |  |  |
|  | Placebo | 96 (50) |  |  |
|  | 28000:28000 IU/week | 96 (50) | -0.053 (-0.21, 0.10) | 0.504 |
| 3'SL | **HMO secretor status** |  |  |  |
|  | No | 65 (34) |  |  |
|  | Yes | 127 (66) | 0.567 (0.41, 0.72) | <0.001 |
|  | **Treatment allocation group** |  |  |  |
|  | Placebo | 96 (50) |  |  |
|  | 28000:28000 IU/week | 96 (50) | 0.055 (-0.09, 0.20) | 0.463 |
| DFLac | **HMO secretor status** |  |  |  |
|  | No | 65 (34) |  |  |
|  | Yes | 127 (66) | 3.145 (2.96, 3.33) | <0.001 |
|  | **Treatment allocation group** |  |  |  |
|  | Placebo | 96 (50) |  |  |
|  | 28000:28000 IU/week | 96 (50) | -0.012 (-0.18, 0.16) | 0.894 |
| 6'SL | **HMO secretor status** |  |  |  |
|  | No | 65 (34) |  |  |
|  | Yes | 127 (66) | -0.102 (-0.23, 0.03) | 0.128 |
|  | **Treatment allocation group** |  |  |  |
|  | Placebo | 96 (50) |  |  |
|  | 28000:28000 IU/week | 96 (50) | -0.018 (-0.14, 0.11) | 0.771 |
| LNT | **HMO secretor status** |  |  |  |
|  | No | 65 (34) |  |  |
|  | Yes | 127 (66) | -0.410 (-0.56, -0.26) | <0.001 |
|  | **Treatment allocation group** |  |  |  |
|  | Placebo | 96 (50) |  |  |
|  | 28000:28000 IU/week | 96 (50) | 0.007 (-0.13, 0.15) | 0.927 |
| LNFP1 | **HMO secretor status** |  |  |  |
|  | No | 65 (34) |  |  |
|  | Yes | 127 (66) | 2.637 (2.40, 2.88) | <0.001 |
|  | **Treatment allocation group** |  |  |  |
|  | Placebo | 96 (50) |  |  |
|  | 28000:28000 IU/week | 96 (50) | -0.006 (-0.23, 0.22) | 0.956 |
| LNFP2 | **HMO secretor status** |  |  |  |
|  | No | 65 (34) |  |  |
|  | Yes | 127 (66) | -0.785 (-0.98, -0.59) | <0.001 |
|  | **Treatment allocation group** |  |  |  |
|  | Placebo | 96 (50) |  |  |
|  | 28000:28000 IU/week | 96 (50) | 0.058 (-0.13, 0.25) | 0.541 |
| LNFP3 | **HMO secretor status** |  |  |  |
|  | No | 65 (34) |  |  |
|  | Yes | 127 (66) | -0.425 (-0.53, -0.32) | <0.001 |
|  | **Treatment allocation group** |  |  |  |
|  | Placebo | 96 (50) |  |  |
|  | 28000:28000 IU/week | 96 (50) | -0.033 (-0.14, 0.07) | 0.533 |
| LSTb | **HMO secretor status** |  |  |  |
|  | No | 65 (34) |  |  |
|  | Yes | 127 (66) | -0.850 (-1.00, -0.70) | <0.001 |
|  | **Treatment allocation group** |  |  |  |
|  | Placebo | 96 (50) |  |  |
|  | 28000:28000 IU/week | 96 (50) | -0.001 (-0.14, 0.14) | 0.990 |
|  | **HMO secretor status** |  |  |  |
| LSTc | No | 65 (34) |  |  |
|  | Yes | 127 (66) | 0.475 (0.31, 0.64) | <0.001 |
|  | **Treatment allocation group** |  |  |  |
|  | Placebo | 96 (50) |  |  |
|  | 28000:28000 IU/week | 96 (50) | -0.074 (-0.23, 0.08) | 0.341 |
|  | **HMO secretor status** |  |  |  |
| DFLNT | No | 65 (34) |  |  |
|  | Yes | 127 (66) | 0.770 (0.51, 1.03) | <0.001 |
|  | **Treatment allocation group** |  |  |  |
|  | Placebo | 96 (50) |  |  |
|  | 28000:28000 IU/week | 96 (50) | -0.060 (-0.30, 0.18) | 0.626 |
|  | **HMO secretor status** |  |  |  |
| LNH | No | 65 (34) |  |  |
|  | Yes | 127 (66) | 0.143 (-0.07, 0.36) | 0.186 |
|  | **Treatment allocation group** |  |  |  |
|  | Placebo | 96 (50) |  |  |
|  | 28000:28000 IU/week | 96 (50) | 0.061 (-0.14, 0.26) | 0.547 |
|  | **HMO secretor status** |  |  |  |
| DSLNT | No | 65 (34) |  |  |
|  | Yes | 127 (66) | -0.045 (-0.18, 0.09) | 0.528 |
|  | **Treatment allocation group** |  |  |  |
|  | Placebo | 96 (50) |  |  |
|  | 28000:28000 IU/week | 96 (50) | -0.065 (-0.20, 0.07) | 0.335 |
|  | **HMO secretor status** |  |  |  |
| FLNH | No | 65 (34) |  |  |
|  | Yes | 127 (66) | 0.862 (0.59, 1.13) | <0.001 |
|  | **Treatment allocation group** |  |  |  |
|  | Placebo | 96 (50) |  |  |
|  | 28000:28000 IU/week | 96 (50) | 0.249 (-0.01, 0.50) | 0.056 |
|  | **HMO secretor status** |  |  |  |
| DFLNH | No | 65 (34) |  |  |
|  | Yes | 127 (66) | 0.701 (0.49, 0.91) | <0.001 |
|  | **Treatment allocation group** |  |  |  |
|  | Placebo | 96 (50) |  |  |
|  | 28000:28000 IU/week | 96 (50) | -0.051 (-0.25, 0.15) | 0.617 |
|  | **HMO secretor status** |  |  |  |
| FDSLNH | No | 65 (34) |  |  |
|  | Yes | 127 (66) | -1.003 (-1.26, -0.75) | <0.001 |
|  | **Treatment allocation group** |  |  |  |
|  | Placebo | 96 (50) |  |  |
|  | 28000:28000 IU/week | 96 (50) | 0.057 (-0.18, 0.30) | 0.641 |
|  | **HMO secretor status** |  |  |  |
| DSLNH | No | 65 (34) |  |  |
|  | Yes | 127 (66) | -0.432 (-0.64, -0.22) | <0.001 |
|  | **Treatment allocation group** |  |  |  |
|  | Placebo | 96 (50) |  |  |
|  | 28000:28000 IU/week | 96 (50) | -0.061 (-0.26, 0.13) | 0.538 |
|  | **HMO secretor status** |  |  |  |
| Total HMO | No | 65 (34) |  |  |
|  | Yes | 127 (66) | 0.659 (0.63, 0.69) | <0.001 |
|  | **Treatment allocation group** |  |  |  |
|  | Placebo | 96 (50) |  |  |
|  | 28000:28000 IU/week | 96 (50) | 0.007 (-0.02, 0.03) | 0.584 |

**Supplementary Figures**


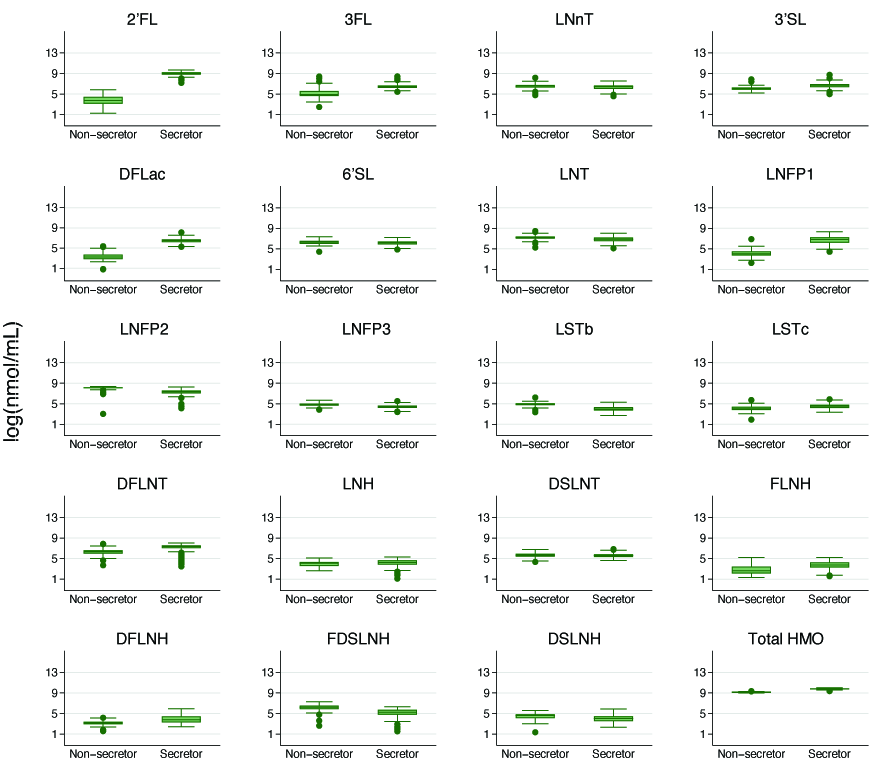


**Supplemental Figure 1**. Total and individual abundances of 19 HMOs among 192 Bangladeshi participants, by secretor status. Boxes represent the interquartile range (IQR) of the absolute concentration of each individual HMO. Whiskers (horizontal lines) indicated ± 1.5 times the IQR. Outliers (i.e., values that are above or below ± 1.5 times the IQR) are shown as filled green circles
